# Effects of Serum Lipid Smoothness on the Progression and Vulnerability of Atherosclerotic Plaques in Rabbits

**DOI:** 10.1371/journal.pone.0093686

**Published:** 2014-07-09

**Authors:** Xiao Zou, Haijun Wang, Lili Cai, Kailiang Li, Wei Zhang, Yu Ding, Quanjin Si

**Affiliations:** 1 Department 1 of the Geriatric Cardiology, Chinese PLA General Hospital, Beijing, P.R. China; 2 Clinical Laboratory of Nanlou, Chinese PLA General Hospital, Beijing, P.R. China; Brigham and Women's Hospital, Harvard Medical School, United States of America

## Abstract

**Objective:**

We aimed to explore the effects of lipid smoothness on the progression and vulnerability of atherosclerotic plaques.

**Approach:**

24 rabbits were divided into three groups randomly. Group 1 was given standard chow diet; group 2 was fed with cholesterol-rich diet; for group 3, subjects were planned to take cholesterol-rich diet at the first phase for 12 weeks and during the second phase, low-fat and cholesterol-rich diet was then applied alternately every three weeks till the end of the experiment. Lipid profiles, inflammatory factors, endothelium functions, pathological and histological changes were examined. Expressions of matrix metalloproteinase-9 and lectin-like oxidized LDL receptor-1 were measured by immunohistochemical staining.

**Results:**

According to data collected during the whole experiment, lipid smoothness index of group 3 was the lowest. Compared with group 2, statistics of the group 3 indicated that: the development of plaques progressed faster; the plaque area and plaque thickness (53.53[22.6]% vs 33.90[24.91]% , 800.38[98.25]µm vs 675.00[109.67]µm) were higher while the fibrous cap thickness (103.50[45.66]µm vs 295.83[97.90]µm) was lower; hs-CRP (0.53[0.07]mg/dL vs 0.45[0.06]mg/dL), interleukin-18 (186.01[8.41]ng/L vs 158.08[2.37]ng/L), OX-LDL (177.15[5.93]µg/L vs 139.57[2.35] µg/L) and endothelin-1 (164.66[9.54]ng/L vs 131.52[4.39]ng/L) were higher while nitric-oxide (22.41[1.69]µmol/L vs 27.23[1.36]µmol/L) was lower; expressions of matrix metalloproteinase-9 (IOD: 37375.87[5634.52] vs 20956.57[4616.93]) and lectin-like oxidized LDL receptor-1 (IOD: 45213.04[16653.81] vs 21921.68[6142.32]) were higher.

**Conclusions:**

Lipids fluctuation could accelerate the progression and vulnerability of atherosclerotic plaques through worsening arterial endothelium dysfunction and inflammatory reactions.

## Introduction

Atherosclerosis is the pathological basis of coronary heart diseases [1], [2]. Rupture and its subsequent thrombosis of the vulnerable atherosclerotic plaque are responsible for the majority of acute coronary events [3]. It is clear that hyperlipidemia could cause atherosclerotic plaque formation and rupture. However, level of some patients' low density lipoprotein cholesterol (LDL-C) has been not elevated or even lower when experiencing acute coronary syndrome (ACS), and those patients were previously diagnosed dyslipidemia. Currently, it is unknown whether the fluctuation of LDL-C level is one of the factors promoting the progression and vulnerability of atherosclerotic plaques. We performed the experiment to find out the effects of lipid smoothness on the progression and vulnerability of atherosclerotic plaques in rabbits.

## Materials and Methods

### 1 Experimental design

This study was carried out in strict accordance with the recommendations in the Guide for the Care and Use of Laboratory Animals of the National Institutes of Health. The protocol was approved by the Committee on the Ethics of Animal Experiments of the University of PLA General Hospital (Permit Number: SCXK [Beijing] 2007–0003). All surgery was performed under sodium pentobarbital anesthesia, and all efforts were made to minimize suffering.

24 male New Zealand White rabbits (2.0–2.5 kg, 12 weeks old) were randomized into three groups. Group 1 was the control group including 6 rabbits, group 2 and 3 were the study groups including 9 rabbits in each. Group 1 was given standard chow diet; group 2 was provided with cholesterol-rich diet containing 1% cholesterol and 3% lard oil during the whole study; for group 3, subjects were planned to take cholesterol-rich diet at the first phase for 12 weeks and during the second phase, low-fat (cholesterol-free diet in granular form) [4] and cholesterol-rich diet was then applied alternately every three weeks till the end of the experiment. Rabbits of group 3 were fed with cholesterol-rich diet in the last three weeks. Each rabbit was raised in a 12h light/dark cycle and temperature-controlled cage seprately, and was given 120gram-designed diet per day, with free access to water. The total experiment lasted 24 weeks.

### 2 Analysis of the serum lipoprotein profile

Blood samples were collected via auricular vein after a period of overnight fasting at week 0, 12^th^, 15^th^, 18^th^, 21^th^ and 24^th^ week. Samples were centrifuged (4000 rpm, 15 minutes, 4°C) to obtain blood serum and then stored in a refrigerator(−80°C). Serum total cholesterol (TC), triglyceride (TG), high-density lipoprotein cholesterol (HDL-C) levels and LDL-C levels were measured by immunoturbidimetry with Roche reagent in PLA General Hospital Clinical Laboratory. Lipoprotein smoothness index (SI) was adopted to evaluate lipoprotein fluctuation calculated as 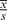 (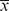 and *s* represented the mean value and standard deviation of lipoprotein profiles from 12^th^ to 24^th^ week respectively). With the SI value lifted, the stability of the lipoprotein level increases.

### 3 Measurement of serum high-sensitivity C-reactive protein (hs-CRP), interleukin-18(IL-18), endothelin-1(ET-1), oxidized low density lipoprotein (OX-LDL) and nitric oxide (NO)

The serum hs-CRP, IL-18, ET-1, OX-LDL and NO levels were measured with rabbit ELISA kits (Santa Cruz Biotechnology, Inc) as previously described in literature [5]–[7].

### 4 Ultrasonography of the abdominal aorta

The abdominal aorta was detected by ultrasound with 8–14MHz transducer (Siemens, SEQUOIA512 type ultrasonic instrument) in 0 week, 12^th^ week and 24^th^ week.

### 5 Pathology analysis

All rabbits were sacrificed, and their aortic trees were isolated and dissected carefully. Both the quantity of gross lesions and the histological changes were analyzed.

For the histological analysis, tissues were stained with Oil Red O and analyzed after fully fixed in 10% formalin as previously described in study [8]. The positively stained areas with Oil Red O were quantified with the Image Pro-PlusV2.0 image analysis software program. Aortic arches were cut into 10 sections, as described [9], for histological analysis. All of the sections were embedded in paraffin and cut into 5 µm-thick serial cross-sectional slices. The gross atherosclerotic lesions were evaluated as previously described in paper [9]. All the aortic sections were stained with hematoxylin-eosin (H&E) for microscopic quantification of the lesion areas. The H&E staining slices were observed with microscope to study the ultrastructure of the atherosclerotic plaque in different groups.

Serial paraffin sections of the thoracic aorta were immunohistochemically stained with antibodies against MMP-9 (matrix metalloproteinase-9) and LOX-1 (lectin-like oxidized low density lipoprotein receptor-1). For the preparations, the sections were washed 3 times with PBS for 5 minutes per wash. Endogenous peroxidases were blocked by incubation for 10 minutes in 0.3% hydrogen peroxide. The sections were washed 3 times with PBS for 5 minutes each. To reduce the background staining, the sections were then incubated in 10% normal goat serum for 60 minutes. The sections were incubated in the primary antibody at 4°C over night, and they were then washed 3 times for 5 minutes with PBS. The secondary antibody (antimurine IgG, Beijing Zhong Shan Biotechnology CO., China) was applied to the sections for 60 minutes. The sections were washed 3 times with PBS for 10 minutes each and were detected with an AEC kit (AEC kit, Beijing Zhong Shan Biotechnology CO., China).

All of the sections (H&E and immunostained) that were used for microscopic quantification were captured under a Motic Olympus BX60 digital camera-applied (Motic Moticcam 2306, Canada) light microscope and were measured using the image pro-plusV2.0 analysis software program (Media Cybernetics Inc., USA). Measurements of plaque thickness and fibrous cap thickness were performed with a length-measurement computer system in which pixels were transformed to micrometers based on a scale set for each image (the image pro-plusV2.0 analysis software) [10].

### 6 Statistical analysis

All data were performed as the mean [SD] unless indicated. Data from each two groups were compared by Student's *t*-test, while three groups were compared by one-way ANOVA. The enumeration data were expressed as frequency and compared with chi-square test. Statistical significance was defined as *P*<0.05. Statistical analysis was performed using the statistical software SPSS ver. 13.0.

## Results

### 1 The general conditions of animals after 24 weeks

At the end of the experiment, one rabbit of group 1 died for unclear reason, one in group 2 and two in group 3 died due to diarrhea. 20 rabbits survived at the end of the experiment. The animals' body weights in all three groups increased, and the animals at 24^th^ week were significantly heavier than 12^th^ week. But there was no statistical significance between groups, see Table 1.

**Table 1:** Body weight in the three groups (mean [SD]).

week	Group 1	Group 2	Group 3
0	2.40[0.16]	2.11[0.22]	2.26[0.22]
12	3.04[0.19]^a^ *	2.98[0.10]^a^ *	3.06[0.15]^a^ *
24	3.45[0.19]^a^ * ^b#^	3.37[0.16]^a^ * b *	3.49[0.13]^a^ * b *

# : *P*<0.05,

*: *P*<0.01,

a : compared with 0 week,

b : compared with week 12.

### 2 Serum lipids profile

**Figure 1:**
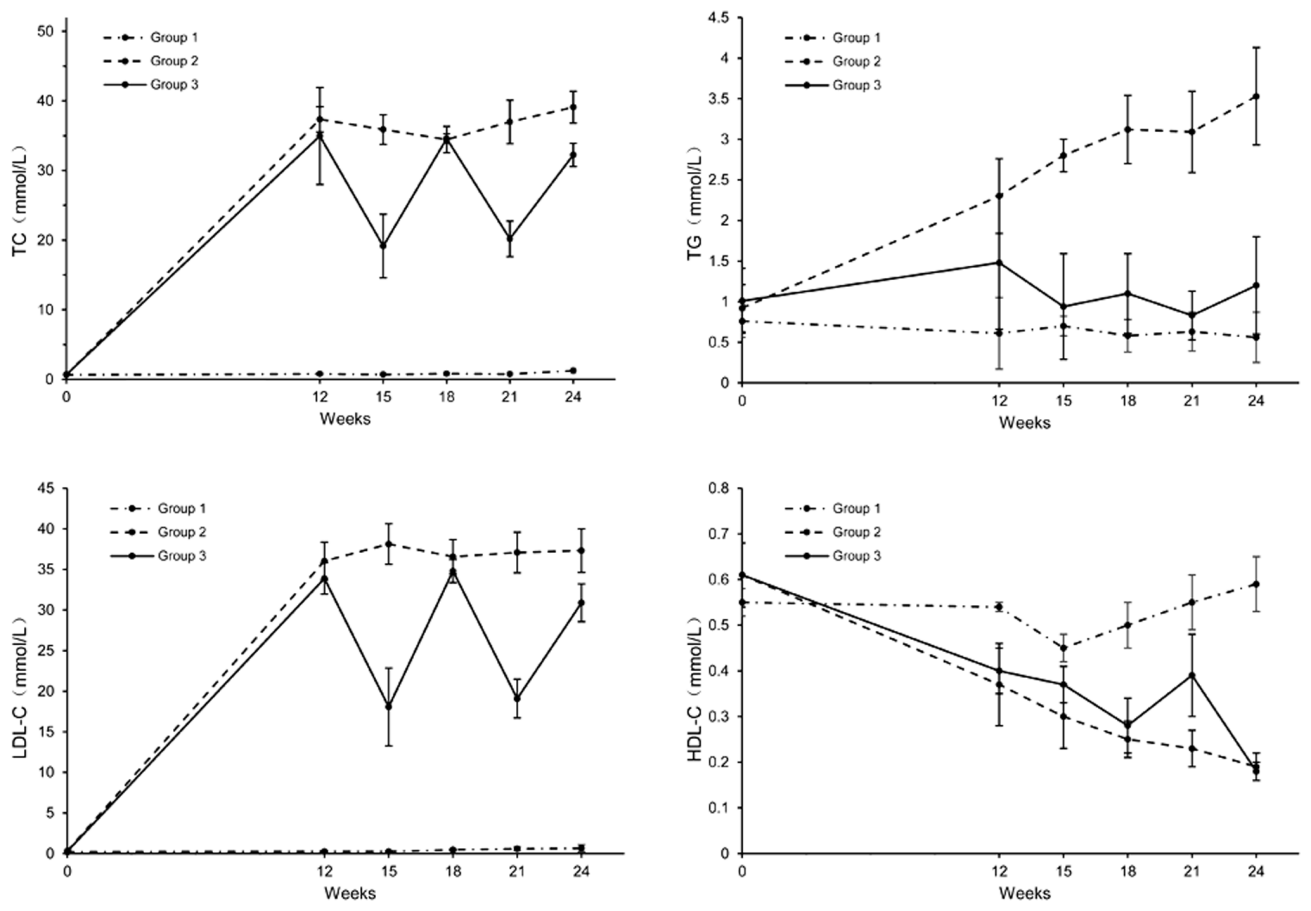
The fluctuations of serum lipids level in the three groups.

Rabbits in group 2 and group 3 all presented severe hypercholesterolemia (Figure 1). Serum HDL-C level decreased dramatically in cholesterol-rich diet fed groups as shown in Figure 1, while TG level did not change compared with the rabbits in group 1 (Figure 1). At 12^th^ week, TC and LDL-C levels in group 2 and 3 were all higher than baseline, also higher than in group 1, and HDL-C level was significantly lower than group 1. TC and LDL-C levels in group 3 were lower than group 2 at 24^th^ week.

The lipids level in group 3 was of significant fluctuation as given cholesterol-rich and low-fat diet alternately. Significant fluctuations were not found in the other two groups (Figure 1).

### 3 Serum lipid smoothness

SI values of each group were calculated at the end of the experiment. As shown in table 2, the SI values in group 2 and 3 were both lower than group 1, and were significant lower in group 3 compared with group 2.

**Table 2:** SI values in the three groups (mean [SD]).

Groups	SI(TC)	SI(LDL-C)
Group 1	20.27[3.84]	9.15[1.16]
Group 2	10.14[0.97]^c^ *	6.73[0.98]^c^ *
Group 3	3.45[0.73]^c^ * d *	3.18[0.77]^c^ * d *

LDL-C: low-density lipoprotein cholesterol, SI: smoothness index, TC: total cholesterol.

*: *P*<0.01,

c : compared with group 1,

d : compared with group 2.

### 4 Changes of serum hs-CRP, IL-18, ET-1, OX-LDL and NO

Hs-CRP, IL-18, ET-1, OX-LDL and NO levels were investigated to evaluate the inflammatory state of the atherosclerotic plaques for all rabbits in three groups. As shown in table 3, hs-CRP, IL-18, OX-LDL and ET-1levels in group 2 and 3 were significantly higher than in group 1 at 12^th^ week, While NO level was significantly lower compared with group 1. The hs-CRP, IL-18, OX-LDL and ET-1 levels kept increasing in group 2 and 3 at 24^th^ week, and they were significantly higher in group 3 than those in group 2. The NO level continued to decline in group 2 and 3 at 24^th^ week, and it was even lower in group 3.

**Table 3:** Serum hs-CRP, IL-18, ET-1, OX-LDL and NO levels in the three groups (mean [SD]).

Items	week	Group 1	Group 2	Group 3
hs-CRP(mg/dL)	0	0.15[0.03]	0.15[0.04]	0.13[0.02]
	12	0.17[0.04]	0.40[0.10]^a^ * c *	0.45[0.11]^a^ * c *
	24	0.15[0.03]	0.45[0.06]^a^ * c *	0.53[0.07]^a^ * ^b#c^ * ^d#^
IL-18(ng/L)	0	134.42[3.87]	134.64[2.80]	133.77[2.23]
	12	136.02[1.49]	158.08[2.37]^a^ * c *	157.55[2.66]^a^ * c *
	24	139.86[1.82]	163.85[2.88]^a^ * b * c *	186.01[8.41]^a^ * b * c * d *
OX-LDL(µg/L)	0	99.15[1.78]	99.63[2.23]	99.67[1.70]
	12	100.43[1.14]	135.08[2.97]^a^ * c *	133.41[3.11]^a^ * c *
	24	101.20[0.65]	139.57[2.35]^a^ * ^b#c^ *	177.15[5.93]^a^ * b * c * d *
NO(µmol/L)	0	42.28[2.29]	40.55[2.59]	42.27[2.96]
	12	41.19[1.11]	33.07[1.73]^a^ * c *	32.25[1.96]^a^ * c *
	24	41.52[1.95]	27.23[1.36]^a^ * b * c *	22.41[1.69]^a^ * b * c * d *
ET-1(ng/L)	0	96.40[2.94]	92.94[6.73]	93.32[4.46]
	12	97.23[2.49]	128.52[5.64]^a^ * c *	129.16[5.26]^a^ * c *
	24	96.51[4.25]	131.52[4.39]^a^ * c *	164.66[9.54]^a^ * b * c * d *

ET-1: endothelin-1, hs-CRP: high-sensitivity C-reactive protein, IL-18: interleukin-18, NO: nitric oxide, OX-LDL: oxidized low density lipoprotein.

# : *P*<0.05,

*: *P*<0.01,

a : compared with 0 week,

b : compared with week 12;

c : compared with group 1,

d :compared with group 2.

### 5 Changes of the aortic ultrasound

**Figure 2:**
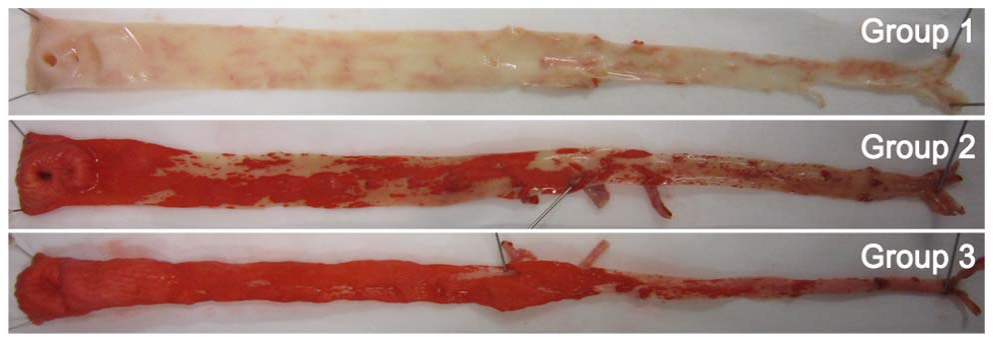
The vascular ultrasound of group 2 and group 3 in different weeks. Legend: (A) and (C): group 2 at week 12 and week 24; (B) and (D): group 3 at week 12 and week 24.

The arterial intima-media thickness (IMT) was severely thickening and multiple plaques were clearly observed in the arterial wall after giving cholesterol-rich diet feeding for 12 weeks. There were no significant differences in IMT between three groups at baseline but it was increased considerably both in group 2 and 3compared with baseline and group 1 at 12^th^ week. IMT thickened greatly at 24^th^ week than 12^th^ week in both group 2 and 3, but changes of IMT in group 3 were more obvious than group 2 at 24^th^ week, see Table 4. Aortic ultrasound showed that the plaque sizes of the same location in group 3 were (0.80×4.90) mm and (1.40×9.90)mm, in group 2 were (0.74×3.46)mm and (0.75×4.34)mm at 12^th^ week and 24^th^ week respectively.(See in Figure 2.)

**Table 4:** The IMT of the three groups in different weeks (mean [SD], mm).

Groups	0 week	week 12	week 24
Group 1	0.39[0.01]	0.40[0.06]	0.41[0.03]
Group 2	0.41[0.04]	0.70[0.11]^a^ * c *	0.77[0.11]^a^ * c *
Group 3	0.43[0.03]	0.84[0.14]^a^ * c *	1.10[0.21]^a^ * ^b#c^ * d *

IMT: intima-media thickness.

# : *P*<0.05,

*: *P*<0.01,

a : compared with 0 week,

b : compared with week 12;

c : compared with group 1,

d : compared with group 2.

### 6 The gross appearance of the aorta

**Figure 3:**
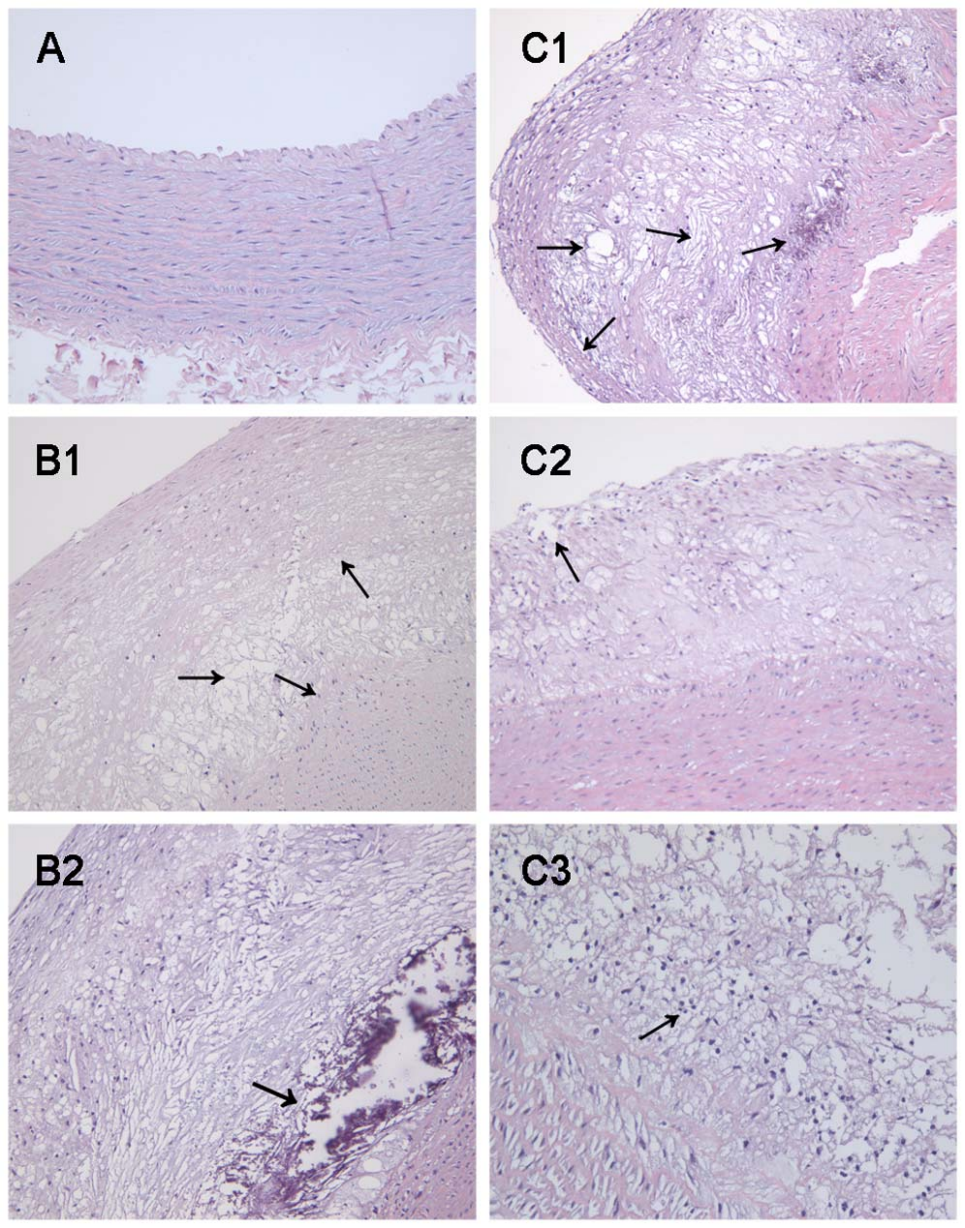
The intima with Oil Red O staining of atherosclerosis in the three groups.

The percentage of plaque area of intima shown as red stained with Oil Red O in group 3 was significantly higher than that in group 2, see Figure 3 and Table 5. Grossly, the intima in group 1 was smooth and was not colored by Oil Red O. Fatty lipid-like material could be observed easily. There were severe atherosclerotic lesions in the vascular intima especially in the aortic arch and the plaques had distinct borders with no surface rupture in group 2. While in group 3, more serious lesions appeared especially in the descending aorta compared with group 2. Diffuse plaques could be seen in the intima and were confluent to big patches. Moreover, the plaque borders were not clear and the surface was not smooth.

**Table 5:** The characteristics of atherosclerotic plaque in the three groups (mean [SD]).

Groups	Percentage of plaque area(%)	Plaque thickness(µm)	Fibrous cap thickness(µm)
Group 1	0	0	0
Group 2	33.90[24.91]^c^ *	675.00[109.67]^c^ *	295.83[97.90]^c^ *
Group 3	53.53[22.60]^c^ * ^d#^	800.38[98.25]^c^ * ^d#^	103.50[45.66]^c^ * d *

# : *P*<0.05,

*: *P*<0.01,

c : compared with group 1,

d : compared with group 2.

### 7 The histological changes of the aorta

**Figure 4:**
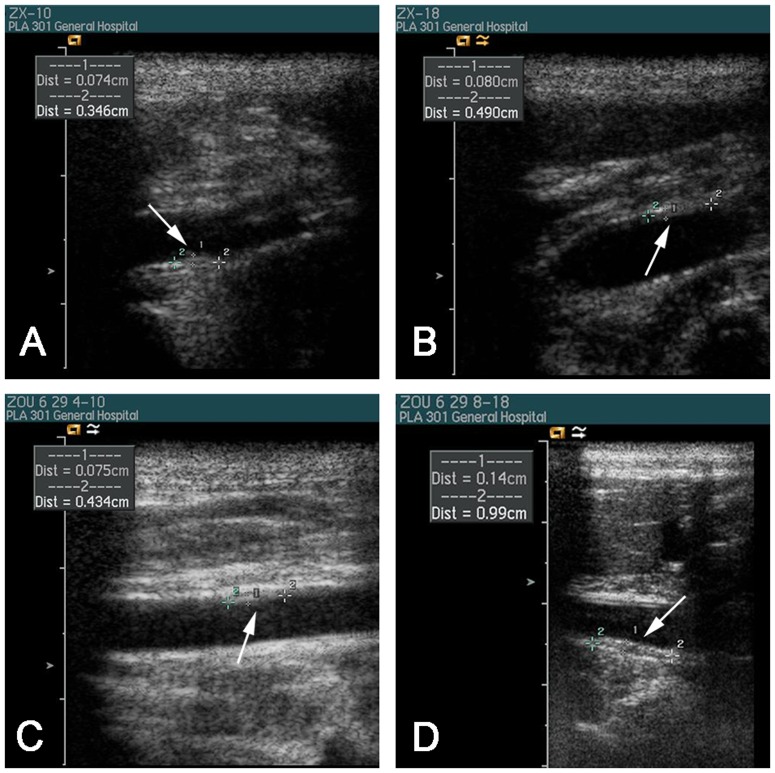
The H&E staining of atherosclerosis in the three groups. Legend: Magnifications: ×200(A–C2), ×400(C3). A: Normal aorta intima in group 1. B1–B2: Atherosclerotic plaque in group 2. The endothelial cells shed, numerous foam cells and cholesterol crystal (B1); cell necrosis and calcium deposition (B2). C1–C3: Atherosclerotic plaque in group 3. plaques with thin fibrous caps and big lipid cores (C1), the discontinuous fiber cap (C2), inflammatory cells (C3).

The aorta intima was smooth with only one layer of endothelial cells without foam cells and lipid accumulation in group 1 under microscopic examination; see Figure 4(A). In group 2, the IMT was obvious thickening and the endothelial cells were shedding. There were obvious atherosclerosis lesions formation with numerous foam cells and cholesterol crystal. Cell necrosis and calcium deposition in some areas also could be seen, see Figure 4(B1–B2). In group 3, atherosclerotic plaques were larger with thinner fibrous caps and bigger lipid cores compared with group 2, see Figure 4(C1). There were amount of inflammatory cells and new vessels in the intima and near the surface of the lesions, and the fiber cap was found discontinuous, as shown in the representative images (Figure 4(C2–C4)). The microscopic lesion thickness of the sections of aortic arch was further calculated. It showed that the lesion thickness in group 3 was significantly thicker than that in group 2 (*P*<0.05). In addition, the fibrous cap thickness was thinner than that in group 2 (*P*<0.01), see Table 5.

### 8 MMP-9 and LOX-1 expressions in aorta

**Figure 5:**
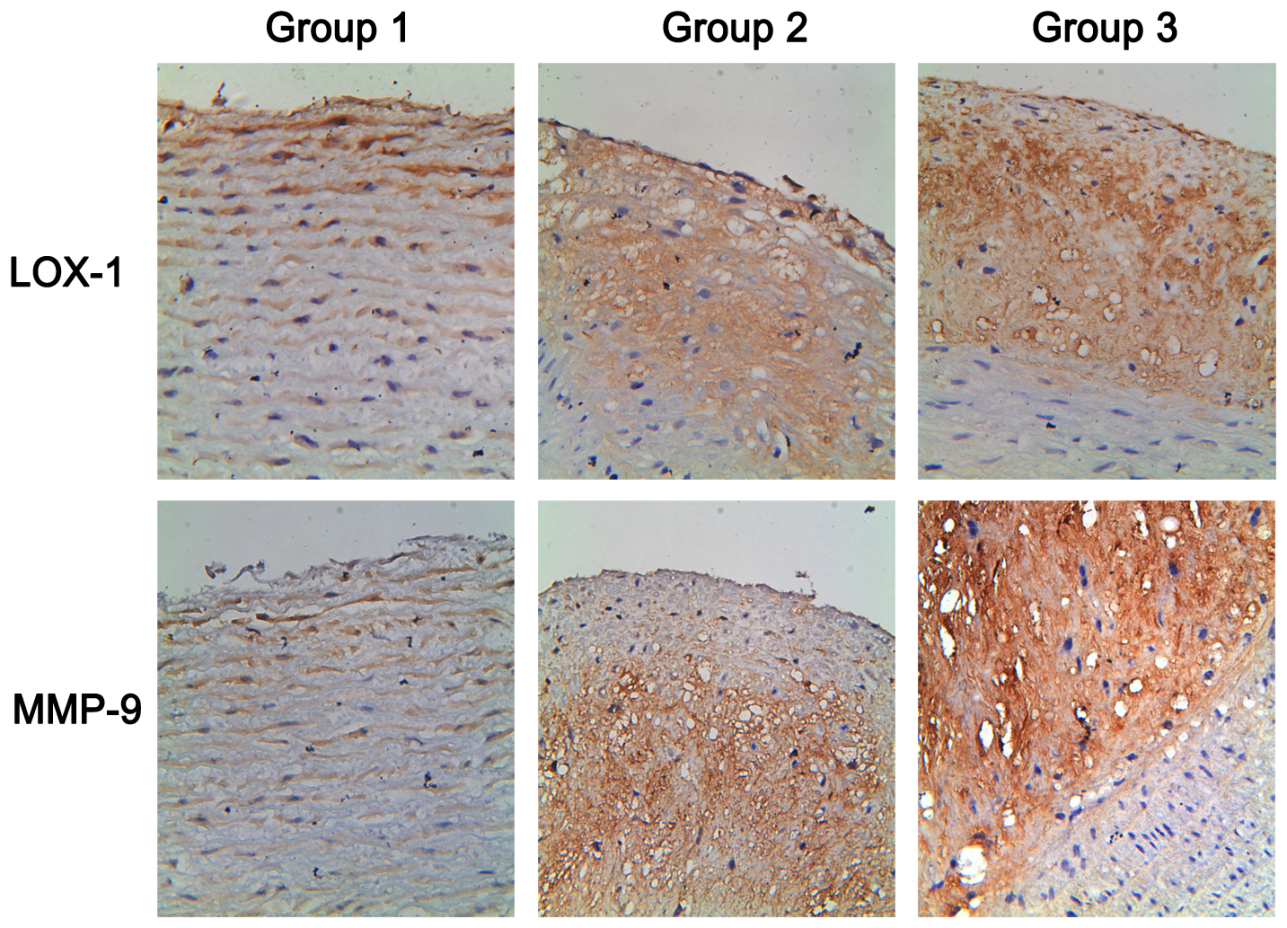
The immuohistochemical staining of atherosclerosis in the 3 groups. Legend: Magnifications: ×400. MMP-9: matrix metalloproteinase-9; LOX-1: lectin-like oxidized low density lipoprotein receptor-1. Representative pictures of MMP-9 and LOX-1 expressions immuohistochemical staining in the aortic atherosclerotic plaque of the three groups.

The immuohistochemical staining of atherosclerosis was performed to detect the expressions of MMP-9 and LOX-1. Compared to group 2, numbers of both MMP-9 and LOX-1-expressing cells were significantly increased in atherosclerotic plaque in group 3(Figure 5). Furthermore, the quantity of MMP-9 and LOX-1 IOD in group 3 were significantly higher than those in group 2, see table 6.

**Table 6:** The IOD of MMP-9 and LOX-1 in the three groups (mean [SD]).

Groups	MMP-9	LOX-1
Group 1	5467.26[1627.64]	3387.85[892.72]
Group 2	20956.57[4616.93]^c^ *	21921.68[6142.32]^c#^
Group 3	37375.87[5634.52]^c^ * d *	45213.04[16653.81]^c^ * d *

IOD: integral optical density, MMP-9: matrix metalloproteinase-9, LOX-1: lectin-like oxidized low density lipoprotein receptor-1.

# : *P*<0.05,

*: *P*<0.01,

a : compared with 0 week,

b : compared with week 12;

c : compared with group 1,

d : compared with group 2.

## Discussion

An abundance of experimental, clinical, and epidemiologic data has shown that hypercholesterolemia was a major causative factor in atherogenesis [11]. Hypercholesterolemia is strongly believed to contribute to atheroma development in human beings and in animal models, and cholesterol exposure directly determined the atherosclerotic progress in the rabbit model [12]. The clear mechanism of ACS is rupture and its subsequent thrombosis of the atherosclerotic plaque following plaque erosion of the unstable atherosclerotic plaques [3]. Plaque rupture is a mechanical event mainly determined by the fibrous cap thickness and the lipid core size. Biological factors such as inflammatory infiltration may contribute to weakening and fracture of the fibrous cap [13]. The vulnerable plaques mainly compose of an atrophic fibrous cap, a lipid-rich necrotic core, and accumulation of inflammatory cells [14]. The main factors involved in the conversion of a stable plaque into an unstable and rupture-prone lesion are hypercholesterolemia [15], vascular endothelial dysfunction [16], as well as the inflammatory response [17], [18]. It is known that a high level of LDL-C is the culprit of atherosclerotic plaque, and its oxidative modified form, ox-LDL, plays an important biological role in the formation of arterial plaque in the early and late phases [19]. In this study, we demonstrated a persistent hyperlipidemia could promote the progression of the atherosclerotic plaques (as in group 2). With previous evidence showed that some ACS patients with hyperlipidemia had a lower level of LDL-C in clinical practice, our study bring to light that lipids fluctuation could also accelerate the atherosclerotic plaque formation and its subsequent vulnerability.

In this study, the rabbit lipids fluctuation model was successfully established by feeding low-fat diet and cholesterol-rich diet every three weeks alternately after the atherosclerotic plaques formation. Lipids SI values were adopted to evaluate the stability of lipid levels for the first time. The results revealed that SI values of LDL-C in group 3 were the lowest but the progression of the atherosclerotic plaques was the fastest between groups, and the smaller the SI values were, the faster of the plaques formation.

Literatures showed that pathological angiogenesis could be seen in atherosclerotic plaques, which might be linked to plaque's progression and subsequent occlusive thrombosis [20]. Pathological angiogenesis provided a pathway infiltrating inflammatory cells into plaques, while plaques infiltrated diffusely by inflammatory cells were always prone to plaque rupture [21], [22]. Some literatures also mentioned that this type of plaque, a thin-capped fibroatheroma with increased inflammatory cell content, was considered to be most vulnerable to disruption [23]. Accordingly, present result showed that the arterial IMT was significantly higher, the plaque area was bigger and the atherosclerotic lesions formation was quicker under ultrasonography in group 3. Moreover, the atherosclerotic lesions of vascular intima in histological examination were significantly larger with a large number of infiltrations of inflammatory cells around plaques. Neovascularization were also found in the intima near the surface of the lesions. The fiber cap thickness was thinner and some fiber cap was even discontinuous. These findings indicated that lipids fluctuation was of impact on the progression and vulnerability of atherosclerotic plaques.

Unstable carotid atherosclerotic plaques are characterized by cap rupture, leading to thromboembolism and stroke. MMPs have been implicated in the progression of atherosclerosis and plaque rupture, and MMP-9 was strongly correlated with plaque instability [24]. The OX-LDL receptor LOX-1, mainly expressed at the shoulder of plaques, plays a crucial role in atherosclerosis. It can activate the inflammatory activity and also induce expression and activation of MMPs [25]. The pathological effects of LOX-1 not only initiate atherosclerotic lesion formation, but also contribute to the vulnerability of a plaque [26]. In this study, expressions of MMP-9 and LOX-1 in group 3 were dramatically higher than in group 2 and group 1. Combining the ultrasonographic and histological changes of atherosclerosis, we suggested that lipids fluctuation might increase the expressions of MMP-9 and LOX-1, accordingly accelerate the progression of atherosclerosis and lead to the formation of unstable plaques.

Previous studies revealed that the development of plaques was closely correlated to the inflammatory process involving the arterial wall [27]. Many literatures supported that inflammation played a pivotal role in all phases of atherosclerosis, from the fatty streak lesion formation to the acute coronary event due to vulnerable plaque rupture [28]. Vascular inflammation contributes to the pathogenesis of atherosclerosis and later in the disease process, it is a major determinant for the acute coronary syndromes. Hs-CRP, a simple downstream marker of inflammation, has been proved to closely connect the atherosclerotic plaque progression [29], [30]. Some studies also showed that thin cap fibroatheromas were commonly seen in those patients with elevated high levels of hs-CRP [13]. Elevated baseline concentrations of hs-CRP are associated with the risk of atherosclerotic events in general population and show a predictive value even in terms of secondary prevention. IL-18 is a pleiotropic proinflammatory cytokine and plays a central role in the inflammatory cascade [31]. It was identified as an interferon-γ–inducing factor, stimulating interferon-γ production in T lymphocytes and natural killer cells, which are ascribed a key role in atherosclerotic plaque growth and vulnerability [32]. This study showed that hs-CRP and IL-18 levels in group 3 were significantly higher than those in the other two groups and therefore, inflammation might play an important role in the accelerating process of atherosclerotic plaques due to lipid fluctuation.

NO and ET-1 level disorder are also key point to promote atherosclerosis progression. In our study, NO level was significant lower while ET-1 level was higher in group 3 than the other groups. Therefore, the endothelium dysfunction of group 3 exacerbated and accelerated the plaque progression. It indicated that the accelerated progression and vulnerability of arterial atherosclerotic plaques caused by instability of lipid levels was mainly linked to activation and worsening of arterial endothelium dysfunction and inflammatory reactions.

In conclusion, marked serum lipids fluctuation is considered to be associated with the atherosclerotic plaque progression and vulnerability in cholesterol-rich diet induced hypercholesterolemia in rabbits. It could exacerbate the arterial endothelium dysfunction and inflammatory reactions, accelerate the progression and impair the stability of atherosclerotic plaques. Our findings might shed light on further exploration of risk factors affecting the atherosclerotic plaque progression and vulnerability in human beings.

In our study, the establishment of rabbit lipid fluctuation model and the idea of observation of the relationship between atherosclerosis progression and lipids fluctuation are novel. There must be some limitations in this study: 1) the sample size of rabbits is not big enough; 2) whether the current animal results can represent the clinical patients or not; 3) the specific mechanisms of lipids fluctuation affecting atherosclerotic plaques. We will continue focusing on the following research in the future.

## References

[1] Libby P, Aikawa M (2002) Stabilization of atherosclerotic plaques: new mechanisms and clinical targets. Nat Med 8(11): 1257–1262. 12411953 10.1038/nm1102-1257

[2] Wu G, Xie Q, Xu L, Jiang H, Huang Z, et al (2013) Pravastatin Inhibits Plaque Rupture and Subsequent Thrombus Formation in Atherosclerotic Rabbits with Hyperlipidemia. Chem Pharm Bull 61(2): 121–124. 23207681 10.1248/cpb.c12-00462

[3] Virmani R, Kolodgie FD, Burke AP, Farb A, Schwartz SM (2000) Lessons from sudden coronary death: a comprehensive morphological classification scheme for atherosclerotic lesions. Arterioscler Thromb Vasc Biol 20(5): 1262–1275. 10807742 10.1161/01.atv.20.5.1262

[4] Juhel C, Pafumi Y, Senft M, Lafont H, Lairon D (2000) chronically gorging v. nibbling fat and cholesterol increases postprandial lipaemia and atheroma deposition in the New Zealand White rabbit. Bri J Nutr 83(5): 549–559. 10953680

[5] Li T, Chen W, An F, Tian H, Zhang J, et al (2011) Probucol attenuates inflammation and increases stability of vulnerable atherosclerotic plaques in rabbits. Tohoku J Exp Med 225(1): 23–34. 21852751 10.1620/tjem.225.23

[6] Wilbert-Lampen U, Trapp A, Barth S, Plasse A, Leistner D (2007) Effects of beta-endorphin on endothelial/monocytic endothelin-1 and nitric oxide release mediated by mu1-opioid receptors: a potential link between stress and endothelial dysfunction? Endothelium 14(2): 65–71. 17497362 10.1080/10623320701346585

[7] Itabe H (1998) Oxidized phospholipids as a new landmark in atherosclerosis. Prog Lipid Res 37(2–3): 181–207. 9829125 10.1016/s0163-7827(98)00009-5

[8] Razavian M, Tavakoli S, Zhang J, Nie L, Dobrucki LW, et al (2011) Atherosclerosis Plaque heterogeneity and response to therapy detected by in vivo molecular imaging of matrix metalloproteinase activation. J Nucl Med 52(11): 1795–1802. 21969358 10.2967/jnumed.111.092379 PMC3235922

[9] Zhao S, Zhang C, Lin Y, Yang P, Yu Q, et al (2008) The effects of rosiglitazone on aortic atherosclerosis of cholesterol-fed rabbits. Thromb Res 123(2): 281–287. 18561986 10.1016/j.thromres.2008.04.011

[10] Silvello D, Narvaes LB, Albuquerque LC, Forgiarini LF, Meurer L, et al. (2013 Dec 26) Serum levels and polymorphisms of matrix metalloproteinases (MMPs) in carotid artery atherosclerosis: higher MMP-9 levels are associated with plaque vulnerability. Biomarkers [Epub ahead of print]. 10.3109/1354750X.2013.866165 24369095

[11] Steinberg D (2005) Hypercholesterolemia and inflammation in atherogenesis: two sides of the same coin. Mol Nutr Food Res 49(11): 995–998. 16270285 10.1002/mnfr.200500081

[12] Yu Q, Li Y, Waqar AB, Wang Y, Huang B, et al (2012) Temporal and quantitative analysis of atherosclerotic lesions in diet-induced hypercholesterolemic rabbits. J Biomed Biotechnol 2012: 506159. 22505812 10.1155/2012/506159 PMC3312324

[13] Fernández Ortiz A (1999) Physiopathology of unstable angina: The role of atherosclerotic plaque rupture and thrombosis. Rev Esp Cardiol 52 (Suppl 1): 3–12 [Article in Spanish]. 10364809

[14] Bea F, Blessing E, Bennett B, Levitz M, Wallace EP, et al (2002) Simvastatin promotes atherosclerotic plaque stability in apoE-deficient mice independently of lipid lowering. Arterioscler Thromb Vasc Biol 22(11): 1832–1837. 12426212 10.1161/01.atv.0000036081.01231.16

[15] Virmani R, Burke AP, Farb A, Kolodgie FD (2006) Pathology of the vulnerable plaque. J Am Coll Cardiol 47 (8 Suppl): C13–C18. 10.1016/j.jacc.2005.10.065 16631505

[16] Rai S, Hare DL, Zulli A (2009) A physiologically relevant atherogenic diet causes severe endothelial dysfunction within 4 weeks in rabbit. Int J Exp Pathol 90(6): 598–604. 19758419 10.1111/j.1365-2613.2009.00668.x PMC2803250

[17] Alekperov ÉZ, Nadzhafov RN (2010) Contemporary concepts of the role of inflammation in atherosclerosis. Kardiologiia 50(6): 88–91. 20659035

[18] Funk SD, Orr AW (2013) Ephs and ephrins resurface in inflammation, immunity, and atherosclerosis. Pharmacol Res 67(1): 42–52. 23098817 10.1016/j.phrs.2012.10.008

[19] Hayashida K, Kume N, Minami M, Kita T (2002) Lectin-like oxidized LDL receptor-1(LOX-1) supports adhesion of mononuclear leukocytes and a monocyte-like cell line THP-1 cells under static and flow conditions. FEBS Lett 511(1–3): 133–138. 11821063 10.1016/s0014-5793(01)03297-5

[20] Ross JS, Stagliano NE, Donovan MJ, Breitbart RE, Ginsburg GS (2001) Atheroselerosis and cancer: common molecular pathways of disease development and progression. Ann N Y Acad Sci 947(12): 271–292. 11795276

[21] Mauriello A, Sangiorgi G, Fratoni S, Palmieri G, Bonanno E, et al (2005) Diffuse and active inflammation occurs in both vulnerable and stable plaques of the entire coronary tree. J Am Coll Cardiol 45(10): 1585–1593. 15893171 10.1016/j.jacc.2005.01.054

[22] Libby P (2005) Act local, act global: inflammation and the multiplicity of “vulnerable” coronary plaques. J Am Coll Cardiol 45(10): 1600–1602. 15893173 10.1016/j.jacc.2005.02.058

[23] Moreno PR, Lodder RA, Purushothaman KR, Charash WE, O'Connor WN, et al (2002) Detection of lipid pool, thin fibrous cap, and inflammatory cells in human aortic atherosclerotic plaques by near-infrared spectroscopy. Circulation 105(8): 923–927. 11864919 10.1161/hc0802.104291

[24] Heo SH, Cho CH, Kim HO, Jo YH, Yoon KS, et al (2011) Plaque rupture is a determinant of vascular events in carotid artery atherosclerotic disease: involvement of matrix metalloproteinases 2 and 9. J Clin Neurol 7(2): 69–76. 21779294 10.3988/jcn.2011.7.2.69 PMC3131541

[25] Li D, Liu L, Chen H, Sawamura T, Mehta JL (2003) LOX-1 Mediates Oxidized LDL-Induced the Expression and Activation of Matrix Metalloproteinases (MMPs) in Human Coronary Artery Endothelial Cells. Circulation 107: 612–617. 12566375 10.1161/01.cir.0000047276.52039.fb

[26] Li D, Patel AR, Klibanov AL, Kramer CM, Ruiz M, et al (2010) Molecular imaging of atherosclerotic plaques targeted to oxidized LDL receptor LOX-1 by SPECT/CT and magnetic resonance. Circ Cardiovasc Imaging 3(4): 464–472. 20442371 10.1161/CIRCIMAGING.109.896654 PMC2955298

[27] Hansson GK (2005) Inflammation, atherosclerosis, and coronary artery disease. N Engl J Med 352(16): 1685–1695. 15843671 10.1056/NEJMra043430

[28] Calabrò P, Golia E, Yeh ET (2009 Jun) CRP and the risk of atherosclerotic events. Semin Immunopathol 31(1): 79–94. 19415283 10.1007/s00281-009-0149-4

[29] Silva D, Pais de Lacerda A (2012) High-sensitivity C-reactive protein as a biomarker of risk in coronary artery disease. Rev Port Cardiol 31(11): 733–745. 23046630 10.1016/j.repc.2012.02.018

[30] Yu MM, Xu Y, Zhang JH, Wang CH, Wang XC, et al (2010) Total cholesterol content of erythrocyte membranes levels are associated with the presence of acute coronary syndrome and high sensitivity C-reactive protein. Int J Cardiol 145(1): 57–58. 19411120 10.1016/j.ijcard.2009.04.018

[31] Gracie JA, Robertson SE, McInnes IB (2003) Interleukin-18. J Leukoc Biol 73: 213–224. 12554798 10.1189/jlb.0602313

[32] Blankenberg S, Luc G, Ducimetière P, Arveiler D, Ferrières J, et al (2003) PRIME Study Group. Interleukin-18 and the risk of coronary heart disease in European men: the Prospective Epidemiological Study of Myocardial Infarction (PRIME). Circulation 108(20): 2453–2459. 14581397 10.1161/01.CIR.0000099509.76044.A2

